# T-Cell Receptor Repertoire as a Predictor of Immune-Related Adverse Events in Renal Cell Carcinoma

**DOI:** 10.3390/cimb45110561

**Published:** 2023-11-09

**Authors:** Takuro Kobayashi, Masayoshi Nagata, Yoshihiro Ikehata, Yuki Nagashima, Naoya Nagaya, Yan Lu, Shigeo Horie

**Affiliations:** 1 Department of Urology, Graduate School of Medicine, Juntendo University, Tokyo 113-8421, Japan; ta-kobayashi@juntendo.ac.jp (T.K.);; 2 Department of Urology, Shizuoka Hospital, Juntendo University, Shizuoka 410-2211, Japan; 3 Department of Advanced Informatics for Genetic Diseases, Graduate School of Medicine, Juntendo University, Tokyo 113-8421, Japan

**Keywords:** renal cell carcinoma, immune checkpoint inhibitors, repertoire analysis, ipilimumab, nivolumab

## Abstract

Immune checkpoint inhibitors (ICIs) are effective in treating renal cell carcinoma (RCC) but can also cause immune-related adverse events (irAEs). The relationship between irAEs and the T-cell receptor (TCR) repertoire in RCC patients treated with ICIs remains unclear. We analyzed the relationship between the severity and diversity of irAEs and the TCR repertoire in RCC patients who received dual checkpoint inhibitors (ipilimumab + nivolumab). The TCRβ (TRB) repertoires were characterized in peripheral blood samples from six patients with RCC before the initiation of ICI therapy. The diversity and clonality of the TCR repertoire were compared between patients with grade 2 and grade 3 irAEs. The median proportion of top 10 unique reads in the TCR repertoire was significantly higher in grade 3 compared with grade 2 irAEs in RCC patients receiving immune checkpoint inhibitors (grade 2: 0.196%; grade 3: 0.346%; *p* = 0.0038). We provide insight into the relationship between TCR repertoire and irAEs in RCC patients treated with ICIs. TCR repertoire clonality may be associated with the development of irAEs in RCC patients.

## 1. Introduction

Immune checkpoint inhibitors (ICIs) are a fundamental treatment for various types of cancer, including renal cell carcinoma (RCC) [1]. These antibodies target programmed cell death protein 1 (PD-1), its ligand (PD-L1), or cytotoxic T-lymphocyte-associated protein 4 (CTLA-4) [2]. Initially used as a monotherapy, ICIs are now frequently used in combination with other treatments such as vascular endothelial growth factor receptor tyrosine kinase inhibitors (VEGFR-TKIs) or dual checkpoint inhibition (anti-PD-(L)1+anti-CTLA-4) [3]. Notably, a combination of antibody-derived nivolumab and ipilimumab has been established as a treatment option for mRCC patients with intermediate or poor risk, as classified by the International Metastatic Renal Cell Carcinoma Database Consortium (IMDC) [4]. However, these treatments also have the potential to cause severe and life-threatening immune-related adverse events (irAEs).

ICI/VEGFR-TKI combination therapy is often associated with multiple organ involvement, and it has been reported that approximately one-third of all deaths are due to myocarditis, myositis, and neurological events [5]. The overall and grade 3–4 incidence of irAEs occurring with ICI/VEGFR-TKI combination therapy in metastatic RCC (mRCC) is considered to be 38–61% and 9–15%, respectively [6,7,8,9]. Several studies have also reported that 81% of patients treated with a combination of nivolumab and ipilimumab experience treatment-related AEs, with a higher incidence of overall and severe irAEs compared with ipilimumab monotherapy [10,11]. Failure to recognize and promptly treat these events can result in the worsening of symptoms and additional complications [2]. Identifying biomarkers that can predict and support the management of irAEs risk is an important area of research.

Recent research suggests that T-cell receptor (TCR) diversity may be a potential indicator of irAEs in patients undergoing ICI treatment. The TCR is made up of a combination of TCRα (TRA) and -β (TRB) chains, which are found on the surface of T cells [12,13]. Through a process called somatic V(D)-J recombination, TCR genes generate a highly diverse T-cell repertoire, which allows for recognition of a wide range of antigens [14,15]. Studies have utilized next-generation sequencing to analyze the TCRs in peripheral blood to investigate whether TCR diversity can be used to predict prognoses in different types of cancer. The results from these studies have shown that TCR diversity in peripheral blood at baseline may be a useful biomarker for predicting or prognosticating cytotoxic CTLA-4 or PD-1 inhibitors [16,17,18,19]. However, due to the limited number of reported studies and the variations in cancer types and patient characteristics, it is unclear which pretreatment immune status may result in irAEs. Identifying patients with diverse TCR repertoires may help to predict those at higher risk of developing irAEs. This information can be used to improve patient outcomes by identifying patients at high risk of irAEs and adjusting treatment accordingly.

The purpose of this study was to examine the association between TCR repertoires measured from pretreatment blood and irAEs in six RCC patients receiving anti-PD-1 therapy.

## 2. Materials and Methods

### 2.1. Patients

Patients who received dual checkpoint inhibition (ipilimumab + nivolumab) as first-line or adjuvant therapy from April 2021 to June 2022 at Juntendo University (Tokyo, Japan) with RCC were the target of this study. The patients were evaluated at baseline and received intravenous administration of nivolumab (240 mg/body every three weeks) and ipilimumab (1 mg/kg every three weeks) without concurrent chemotherapy. Treatment was given until progressive disease or intolerable toxicity occurred. The severity of adverse events was graded using the National Cancer Institute Common Terminology Criteria for Adverse Events (NCI-CTCAE) version 4.0, and the causal relationship with anti-PD-1 therapy was determined by the principal investigator.

### 2.2. RNA Extraction

All blood samples were taken just prior to starting ipilimumab and nivolumab. Total RNA was extracted from PBMCs using an RNeasy Plus Universal Mini Kit (Qiagen, Hilden, Germany) as per the manufacturer’s instructions. The RNA quantity and purity were measured using an Agilent 4200 TapeStation (Agilent Technologies, Palo Alto, CA, USA).

### 2.3. Unbiased Amplification and Sequencing of TCR Genes

Next-generation sequencing analysis was performed using unbiased TCR repertoire analysis technology developed by Repertoire Genesis Inc. (Osaka, Japan). In brief, unbiased adaptor-ligation PCR was performed as per previous reports, and total RNA was converted to cDNA using Superscript III reverse transcriptase (Invitrogen, Carlsbad, CA, USA) with the BSL-18E primer containing polyT18 and a NotI site (Supplementary Table S1) [20,21]. Double-stranded cDNA was synthesized using *E. coli* DNA polymerase I (Invitrogen), *E. coli* DNA Ligase (Invitrogen), and RNase H (Invitrogen). The cDNA was blunted with T4 DNA polymerase (Invitrogen) and the P10EA/P20EA adaptor was ligated to the 5′ end of the double-stranded cDNA and then cut with the NotI restriction enzyme. After removal of the adaptor and primer using Agencourt AMPure XP (Beckman Coulter, Brea, CA, USA), PCR was performed with KAPA HiFi DNA Polymerase (Kapa Biosystems, Woburn, MA, USA) using constant region-specific 1st PCR and P20EA primers (Supplementary Table S1). The PCR conditions were as follows: 98 °C (20 s), 65 °C (30 s), and 72 °C (1 min) for 20 cycles. The second PCR was performed with 2nd PCR and P20EA primers using the same PCR conditions. Amplicons were prepared by amplifying the second PCR products using Tag PCR and P22EA-ST1-R primers (Supplementary Table S1). After PCR amplification, index (barcode) sequences were added using a Nextera XT Index Kit v2, setA or setD (Illumina, San Diego, CA, USA). The indexed amplicon products were mixed in equal molar concentrations and quantified using a Qubit 3.0 Fluorometer (Thermo Fisher Scientific, Waltham, MA, USA). Sequencing was performed using the Illumina Miseq paired-end platform (2 × 300 bp).

### 2.4. Data Analysis

All paired-end reads were classified according to index sequences. Sequence assignment was performed by determining sequences with the highest identity in a dataset of reference sequences from the International ImMunoGeneTics information system^®^ (IMGT) database (https://www.imgt.org) (accessed on 12 September 2018). Data processing, assignment, and data aggregation were automatically performed using the Repertoire Genesis (RG) software originally developed by Repertoire Genesis Inc. (Osaka, Japan). RG implemented a program for sequence homology searches using BLATN, an automatic aggregation program, a graphics program for gene usage, and complementary determining region 3 (CDR3) length distribution. Sequence identities at the nucleotide level between query and entry sequences were automatically calculated. The parameters that increased sensitivity and accuracy (E-value threshold, minimum kernel, high-scoring segment pair score) were carefully optimized for respective repertoire analysis. The nucleotide sequences of CDR3 regions ranging from conserved cysteine at position 104 (Cys104) of IMGT nomenclature to conserved phenylalanine or tryptophan at position 118 (Phe118 or Trp118) were translated to deduced amino acid sequences. A unique sequence read (USR) was defined as a sequence read with no identity in the assignment of gene segments and a deduced amino acid sequence of CDR3 with other sequence reads. The copy number of identical USRs in each sample was automatically counted by the RG software and then ranked in order of the copy number. Percentage occurrence frequencies of sequence reads with V and J genes in total sequence reads were calculated.

### 2.5. Statistical Analysis and Graph Drawing

The TCR repertoire diversity prior to treatment was evaluated using two different methods: The Shannon–Weaver index and the inverse Simpson’s index. These methods were used to consider the influence of the number of unique receptors (richness) and their relative abundance (evenness). Two-sided *p*-values, evaluated using the Wilcoxon rank-sum test, were used to compare the TCR repertoire diversity or clonality. A two-tailed *p*-value of less than 0.05 was considered statistically significant for all analyses. All statistical analyses were performed using R, version 4.1.2 (R Foundation for Statistical Computing, Vienna, Austria). Circos plots were created using the circlize 0.4.15 package. Heatmaps of V and J frequencies were drawn using heatmap.2 function from gplots 3.1.3 package. All graphs were plotted using ggplot2 3.4.0.

## 3. Results

### 3.1. Patient Characteristics

The median age of the patients was 70.5 years (range, 27–73 years), with a mean age of 63.8 ± 18.1 years. The cohort included three males and three females, and 83.3% (n = 5) had a history of smoking. Of the patients, 66.7% (n = 4) had T3a or a higher tumor size and 66.7% (n = 4) had metastasis. Meanwhile, 33.3% (n = 2) were classified as poor risk by the IMDC criteria, while the rest were intermediate risk. Most of the patients (83.3%, n = 5) had clear-cell RCC, with one patient having a MiT-family translocation. Overall, 50.0% (n = 3) of the patients received ipilimumab + nivolumab as first-line treatment, while the others received as adjuvant therapy. Patient RCC6 underwent laparoscopic nephrectomy followed by partial pulmonary resection for metastatic lesions in the lungs. Subsequent to this, the patient received adjuvant therapy upon recurrence. All patients experienced irAEs, with 50.0% (n = 3) being grade 3. Detailed information about the irAEs is presented in Table 1.

### 3.2. TCR Profile

**Figure 1:**
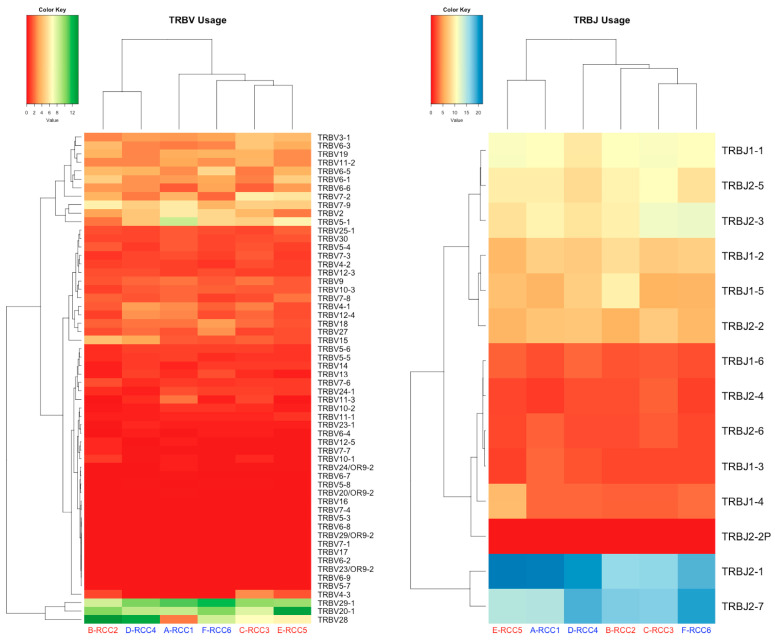
Heatmaps of V (**Left)** and J (**Right**) gene frequencies for TRB in RCC patients’ blood. irAE grade 2 is indicated in blue letters and grade 3 in red letters. TRB: T-cell receptor β chain.

**Figure 2:**
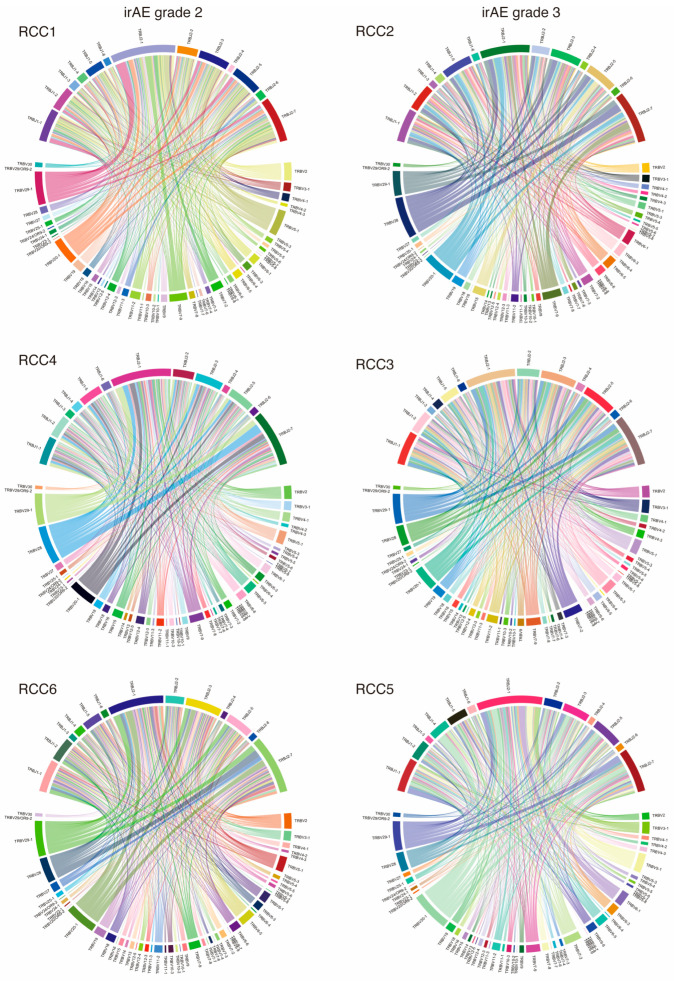
VJ gene combinations and their frequencies in TRB of six patients by circos plot. Each link shows a specific VJ gene combination and its frequency of presence, with thicker links indicating higher frequencies of presence. TRB: T-cell receptor β chain.

We assessed the variation in the TCR immune repertoire in patients between two different irAE groups. In the grade 2 group, the most frequent TRBV/J gene segments of clonal type were TRBV28 (12.70%) and TRBJ2-1 (21.50%) (Figure 1 and Table 2). Meanwhile, the most abundant TRBV/J gene segments of clonal type in grade 3 group were TRBV28 (13.58%) and TRBJ2-1 (21.84%). The frequency of some genes within TRBV and TRBJ differed by grade of irAE (Supplementary Figure S1). In addition, regardless of the grade of irAE, the chronotypes of the Vβ-CDR3-Jβ combinations were diversified in both groups (Figure 2). Full TRBV/J information is in Supplementary Tables S2 and S3.

### 3.3. Association between the irAE Grade and TCR Repertoire Diversity

We evaluated the relationship between the severity and diversity of the irAE using the Shannon–Weaver index (S-W) and the inverse Simpson’s index (Inv.Simpson) (Table 3). The median S-W index for grade 2 and grade 3 groups was 9.39 (8.92–9.50) and 8.91 (8.03–10.34), respectively (Supplementary Table S4). Additionally, the median Inv.Simpson values for grade 2 and grade 3 groups were 2607 (1751–6672) and 1159.2 (806.0–5394.4), respectively. Grade 3 group had a lower diversity than grade 2, but no significant difference was found between the two groups in terms of these two indices (S-W: *p* = 0.70; Inv.Simpson: *p* = 0.40).

### 3.4. Association between the irAE Grade and TCR Repertoire Clonality

**Figure 3:**
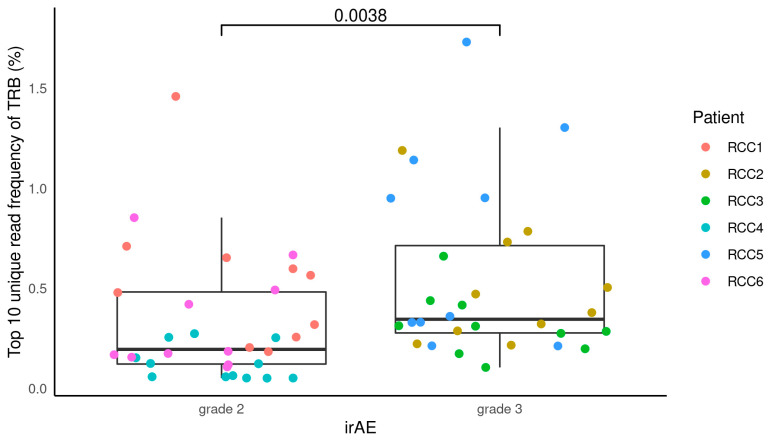
Relationship between the proportion of top 10 unique reads and the severity of the irAE. Each plot shows the top 10 unique reads per patient by color. irAE: immune-related adverse event; TRB: T-cell receptor β chain.

Furthermore, we looked at the relationship between the proportion of top 10 unique reads and the severity of irAE by analyzing clonality. The grade 2 group had a median proportion of top 10 unique reads of 0.196% (0.05–1.46%), whereas the grade 3 group had a median proportion of 0.35% (0.11–1.73%) (Figure 3 and Supplementary Table S5). The association between clonality and irAE severity was found, with grade 3 showing higher clonality than the grade 2 group (*p* = 0.0038).

## 4. Discussion

It has been difficult to predict the occurrence of irAEs with the use of ICIs in patients with mRCC. In this study, the relationship between the severity and diversity of irAEs and the TCR repertoire was analyzed in six patients with RCC treated with ICIs. The results showed no significant differences in the diversity of the TCR repertoire between patients with grade 2 and grade 3 irAEs, but a significant difference in clonality between the two groups was observed. It is noteworthy that although the differences in expression of the V and J genes and their recombination between RCC patients in the irAE grade 2 and 3 groups were negligible, there were significant differences between the two groups when the top 10 unique reads were examined. Our study suggests that TCR repertoire clonality may have potential implications for identifying patients at high risk of irAEs.

The onset of irAEs has been suggested to be a potential clinical biomarker of the ICI response in patients. In previous studies, patients with stage III/IV non-small-cell lung cancer (NSCLC) who developed irAEs after treatment with anti-PD-(L)1 ICIs alone or in combination with other anticancer agents had better overall and progression-free survival compared to those who did not develop irAEs [22]. Patients who responded to treatment with anti-PD-1/L1 antibodies in a study of patients with metastatic or locally advanced urothelial carcinoma were almost twice as likely to report associated adverse events or associated immune-mediated adverse events [23]. In a retrospective two-center study of patients with metastatic RCC receiving first- or second-line therapy with ICIs, 42.2% of the 90 patients experienced an irAE, and this was associated with improved overall survival and time to next therapy in a multivariate analysis [24]. A retrospective analysis of patients from an Italian database with previously treated metastatic RCC who were treated with nivolumab showed that patients who experienced an irAE had prolonged overall survival compared with those who did not, with a one-year overall survival rate of 75.4% versus 59.8% [25]. mRCC patients who experienced a thyroid irAE in a retrospective study of 200 mRCC patients treated with ICIs at the Winship Cancer Institute from 2015 to 2020 had significantly improved clinical outcomes compared to those who did not [26].

irAEs are thought to be caused by activated T cells, but the mechanisms behind specific toxicities in specific patients, and the link between the severity of irAEs and the response are not yet clear [27,28]. Several studies have suggested that irAEs are caused by antigens common to both the tumor and inflammatory organs. In a postmortem study of a patient with metastatic melanoma who developed fulminant myocarditis with rhabdomyolysis after combination therapy with nivolumab and ipilimumab, T-cell and macrophage infiltration was found in the myocardium, and TCR sequencing of infiltrating T cells revealed a high TCR common to the tumor and skeletal muscle [29]. In a prospective cohort study of 73 NSCLC patients treated with anti-PD-1 antibodies, 34.2% of patients developed cutaneous irAEs, and TCR chronotyping of samples from four patients with matched skin and tumor biopsies revealed the presence of a T-cell clone shared between the skin and tumor in all patients [30]. In other studies, TCRα and β repertoires were characterized in peripheral blood samples from 25 patients with advanced RCC before and one, three, and six months after the initiation of PD-1 therapy [19]. The TCR repertoire analysis revealed that those who responded to anti-PD-1 treatment also had specific T-cell clone expansion, with a significant decrease in the TCR diversity index after one month of treatment [19]. These studies suggest that changes in the TCR repertoire may be associated with the response to anti-PD-1 therapy in patients with advanced RCC.

There are several limitations to this study. Firstly, the sample size of this study was small (n = 6), and it primarily consisted of patients from specific irAE grades. This lack of diversity within the small cohort might have influenced the outcomes and our capacity to generalize the findings across a broader RCC patient population. As a result, our conclusions should be interpreted with caution. While previous research has suggested that TCR diversity could be a potential indicator of irAEs in patients undergoing ICI treatment, our findings did not support this hypothesis. Secondly, the relationship between irAEs and prognosis is complex and may vary depending on the specific ICI being used. Thirdly, the absence of a control group, especially related family members, is another limitation. Factors such as environmental pathogens, allergens, and seasonal changes can affect TCR repertoire dynamics. Moreover, our cohort, being exclusively of Japanese descent, did not undergo HLA allele analysis. HLA variations, known to impact T-cell responses, can lead to diverse T-cell clones in different individuals. Including both healthy and diseased controls would have enriched our understanding of TCR repertoire shifts. Lastly, focusing only on the TCRβ repertoire without the paired TCRα data impedes a comprehensive understanding of T-cell clonality and specificity. The combined insights from both chains are vital for a thorough grasp of the T-cell response.

## 5. Conclusions

In conclusion, the onset of irAEs has been suggested to be a potential clinical biomarker of the ICI response in patients. The results of this study suggest that TCR repertoire clonality may have potential implications for identifying patients at high risk of irAEs. The identification of patients at higher risk of developing irAEs is crucial for optimizing patient outcomes. By recognizing those with a higher likelihood of experiencing severe irAEs, clinicians can adjust treatment strategies accordingly, such as closely monitoring patients, providing timely intervention, or considering alternative therapies. Our study contributes to this effort by highlighting the potential utility of TCR clonality as a predictive biomarker for irAE risk in RCC patients receiving anti-PD-1 therapy. Further research is needed to better understand the relationship between irAEs and the response and to determine the optimal way to use irAEs as a predictor of treatment outcome.

## Figures and Tables

**Table 1:** Patient characteristics.

Characteristics	RCC1	RCC2	RCC3	RCC4	RCC5	RCC6
Age, years	73	73	71	27	70	69
Sex	Female	Female	Male	Female	Male	Male
Smoking	Yes	Yes	Yes	No	Yes	Yes
Stage						
T	3a	1b	3a	3a	3a	1b
N	0	0	1	1	0	0
M	1	1	1	0	0	1
IMDC risk	intermediate	intermediate	poor	intermediate	intermediate	poor
Pathology	Clear-cell RCC	Clear-cell RCC	Clear-cell RCC	MiT-family translocation	Clear-cell RCC	Clear-cell RCC
Treatment lines	First-line	First-line	First-line	Adjuvant	Adjuvant	Adjuvant
irAE						
Grade	2	3	3	2	3	2
Rash	Yes	No	No	Yes	No	Yes
Hepatitis	Yes	No	No	No	No	Yes
Diarrhea/colitis	No	Yes	No	Yes	Yes	No
Hypothyroidism	No	Yes	No	No	No	No
Adrenal insufficiency	No	No	Yes	No	Yes	No

IMDC: International Metastatic Renal Cell Carcinoma Database Consortium; irAE: immune-related adverse event; RCC: renal cell carcinoma.

**Table 2:** Top five most frequently used TRB genes by grade of irAE.

TRB Genes	Grade 2			Grade 3		
	Usage Pattern	Frequency (%)	Patient	Usage Pattern	Frequency (%)	Patient
TRBV	TRBV28	12.70	RCC4	TRBV28	13.58	RCC2
	TRBV29-1	11.75	RCC6	TRBV17	12.98	RCC5
	TRBV29-1	11.03	RCC1	TRBV20-1	10.20	RCC2
	TRBV29-1	10.42	RCC4	TRBV7-3	9.93	RCC3
	TRBV20-1	10.27	RCC6	TRBV7-1	9.74	RCC5
TRBJ	TRBJ2-1	21.50	RCC1	TRBJ2-1	21.84	RCC5
	TRBJ2-1	20.11	RCC4	TRBJ2-7	17.13	RCC2
	TRBJ2-7	19.46	RCC6	TRBJ2-7	17.01	RCC3
	TRBJ2-7	18.62	RCC4	TRBJ2-1	16.47	RCC3
	TRBJ2-1	18.43	RCC6	TRBJ2-1	16.32	RCC2

TRB: T-cell receptor β chain.

**Table 3:** TCR diversity by the severity of the irAE.

Diversity Index	Grade 2	Grade 3	*p* Value
Shannon–Weaver index	9.39 (8.92–9.50)	8.91 (8.03–10.34)	0.70
inverse Simpson’s index	2607 (1751–6672)	1159.2 (806.0–5394.4)	0.40

TRB: T-cell receptor β chain.

## Data Availability

The data presented in this study are available on request from the corresponding author.

## Supplementary Materials

The following supporting information can be downloaded at: https://www.mdpi.com/article/10.3390/cimb45110561/s1, Figure S1: TRB usage by grade of irAE; Table S1: Primers used in this study; Table S2: TRBV gene usage in each patient; Table S3: TRBJ gene usage in each patient; Table S4: TRB reads counts and diversity index; Table S5: Top 10 V/J CDR3 usage in TRB for each patient.

## References

[1] Atkins M.B., Tannir N.M. (2018). Current and emerging therapies for first-line treatment of metastatic clear cell renal cell carcinoma. Cancer Treat. Rev..

[2] Martins F., Sofiya L., Sykiotis G.P., Lamine F., Maillard M., Fraga M., Shabafrouz K., Ribi C., Cairoli A., Guex-Crosier Y. (2019). Adverse effects of immune-checkpoint inhibitors: Epidemiology, management and surveillance. Nat. Rev. Clin. Oncol..

[3] Byun D.J., Wolchok J.D., Rosenberg L.M., Girotra M. (2017). Cancer immunotherapy—Immune checkpoint blockade and associated endocrinopathies. Nat. Rev. Endocrinol..

[4] Ljungberg B., Albiges L., Abu-Ghanem Y., Bedke J., Capitanio U., Dabestani S., Fernandez-Pello S., Giles R.H., Hofmann F., Hora M. (2022). European Association of Urology Guidelines on Renal Cell Carcinoma: The 2022 Update. Eur. Urol..

[5] Wang D.Y., Salem J.E., Cohen J.V., Chandra S., Menzer C., Ye F., Zhao S., Das S., Beckermann K.E., Ha L. (2018). Fatal Toxic Effects Associated with Immune Checkpoint Inhibitors: A Systematic Review and Meta-analysis. JAMA Oncol..

[6] Motzer R., Alekseev B., Rha S.Y., Porta C., Eto M., Powles T., Grunwald V., Hutson T.E., Kopyltsov E., Mendez-Vidal M.J. (2021). Lenvatinib plus Pembrolizumab or Everolimus for Advanced Renal Cell Carcinoma. N. Engl. J. Med..

[7] Motzer R.J., Penkov K., Haanen J., Rini B., Albiges L., Campbell M.T., Venugopal B., Kollmannsberger C., Negrier S., Uemura M. (2019). Avelumab plus Axitinib versus Sunitinib for Advanced Renal-Cell Carcinoma. N. Engl. J. Med..

[8] Powles T., Plimack E.R., Soulieres D., Waddell T., Stus V., Gafanov R., Nosov D., Pouliot F., Melichar B., Vynnychenko I. (2020). Pembrolizumab plus axitinib versus sunitinib monotherapy as first-line treatment of advanced renal cell carcinoma (KEYNOTE-426): Extended follow-up from a randomised, open-label, phase 3 trial. Lancet Oncol..

[9] Motzer R.J., Choueiri T.K., Powles T., Burotto M., Bourlon M.T., Hsieh J.J., Maruzzo M., Shah A.Y., Suarez C., Barrios C.H. (2021). Nivolumab + cabozantinib (NIVO + CABO) versus sunitinib (SUN) for advanced renal cell carcinoma (aRCC): Outcomes by sarcomatoid histology and updated trial results with extended follow-up of CheckMate 9ER. J. Clin. Oncol..

[10] Motzer R.J., Rini B.I., McDermott D.F., Aren Frontera O., Hammers H.J., Carducci M.A., Salman P., Escudier B., Beuselinck B., Amin A. (2019). Nivolumab plus ipilimumab versus sunitinib in first-line treatment for advanced renal cell carcinoma: Extended follow-up of efficacy and safety results from a randomised, controlled, phase 3 trial. Lancet Oncol..

[11] Zhou S., Khanal S., Zhang H. (2019). Risk of immune-related adverse events associated with ipilimumab-plus-nivolumab and nivolumab therapy in cancer patients. Ther. Clin. Risk Manag..

[12] Scaviner D., Lefranc M.P. (2000). The human T cell receptor alpha variable (TRAV) genes. Exp. Clin. Immunogenet..

[13] Folch G., Lefranc M.P. (2000). The human T cell receptor beta variable (TRBV) genes. Exp. Clin. Immunogenet..

[14] Davis M.M., Bjorkman P.J. (1988). T-cell antigen receptor genes and T-cell recognition. Nature.

[15] Davis M.M. (1990). T cell receptor gene diversity and selection. Annu. Rev. Biochem..

[16] Kato T., Iwasaki T., Uemura M., Nagahara A., Higashihara H., Osuga K., Ikeda Y., Kiyotani K., Park J.H., Nonomura N. (2017). Characterization of the cryoablation-induced immune response in kidney cancer patients. Oncoimmunology.

[17] Liu Y.Y., Yang Q.F., Yang J.S., Cao R.B., Liang J.Y., Liu Y.T., Zeng Y.L., Chen S., Xia X.F., Zhang K. (2019). Characteristics and prognostic significance of profiling the peripheral blood T-cell receptor repertoire in patients with advanced lung cancer. Int. J. Cancer.

[18] Kato T., Tomiyama E., Koh Y., Matsushita M., Hayashi Y., Nakano K., Ishizuya Y.U., Wang C., Hatano K., Kawashima A. (2020). A Potential Mechanism of Anticancer Immune Response Coincident with Immune-related Adverse Events in Patients with Renal Cell Carcinoma. Anticancer Res..

[19] Kato T., Kiyotani K., Tomiyama E., Koh Y., Matsushita M., Hayashi Y., Nakano K., Ishizuya Y., Wang C., Hatano K. (2021). Peripheral T cell receptor repertoire features predict durable responses to anti-PD-1 inhibitor monotherapy in advanced renal cell carcinoma. Oncoimmunology.

[20] Kitaura K., Shini T., Matsutani T., Suzuki R. (2016). A new high-throughput sequencing method for determining diversity and similarity of T cell receptor (TCR) alpha and beta repertoires and identifying potential new invariant TCR alpha chains. BMC Immunol..

[21] Kitaura K., Yamashita H., Ayabe H., Shini T., Matsutani T., Suzuki R. (2017). Different Somatic Hypermutation Levels among Antibody Subclasses Disclosed by a New Next-Generation Sequencing-Based Antibody Repertoire Analysis. Front. Immunol..

[22] Shankar B., Zhang J., Naqash A.R., Forde P.M., Feliciano J.L., Marrone K.A., Ettinger D.S., Hann C.L., Brahmer J.R., Ricciuti B. (2020). Multisystem Immune-Related Adverse Events Associated with Immune Checkpoint Inhibitors for Treatment of Non-Small Cell Lung Cancer. JAMA Oncol..

[23] Maher V.E., Fernandes L.L., Weinstock C., Tang S., Agarwal S., Brave M., Ning Y.-m., Singh H., Suzman D., Xu J. (2019). Analysis of the Association between Adverse Events and Outcome in Patients Receiving a Programmed Death Protein 1 or Programmed Death Ligand 1 Antibody. J. Clin. Oncol..

[24] Elias R., Yan F., Singla N., Levonyack N., Formella J., Christie A., Kapur P., Bowman A.I., Hammers H.J., Hannan R. (2019). Immune-related adverse events are associated with improved outcomes in ICI-treated renal cell carcinoma patients. J. Clin. Oncol..

[25] Verzoni E., Carteni G., Cortesi E., Giannarelli D., De Giglio A., Sabbatini R., Buti S., Rossetti S., Cognetti F., Rastelli F. (2019). Real-world efficacy and safety of nivolumab in previously-treated metastatic renal cell carcinoma, and association between immune-related adverse events and survival: The Italian expanded access program. J. Immunother. Cancer.

[26] Martini D.J., Goyal S., Liu Y., Evans S.T., Olsen T.A., Case K., Magod B.L., Brown J.T., Yantorni L., Russler G.A. (2021). Immune-Related Adverse Events as Clinical Biomarkers in Patients with Metastatic Renal Cell Carcinoma Treated with Immune Checkpoint Inhibitors. Oncologist.

[27] Yoest J.M. (2017). Clinical features, predictive correlates, and pathophysiology of immune-related adverse events in immune checkpoint inhibitor treatments in cancer: A short review. Immunotargets Ther..

[28] Passat T., Touchefeu Y., Gervois N., Jarry A., Bossard C., Bennouna J. (2018). Physiopathological mechanisms of immune-related adverse events induced by anti-CTLA-4, anti-PD-1 and anti-PD-L1 antibodies in cancer treatment. Bull. Cancer.

[29] Johnson D.B., Balko J.M., Compton M.L., Chalkias S., Gorham J., Xu Y., Hicks M., Puzanov I., Alexander M.R., Bloomer T.L. (2016). Fulminant Myocarditis with Combination Immune Checkpoint Blockade. N. Engl. J. Med..

[30] Berner F., Bomze D., Diem S., Ali O.H., Fassler M., Ring S., Niederer R., Ackermann C.J., Baumgaertner P., Pikor N. (2019). Association of Checkpoint Inhibitor-Induced Toxic Effects with Shared Cancer and Tissue Antigens in Non-Small Cell Lung Cancer. JAMA Oncol..

