# Supplementary figures and images for: T-Cell Receptor Repertoire as a Predictor of Immune-Related Adverse Events in Renal Cell Carcinoma
Source: Curr Issues Mol Biol. 2023 Nov 9;45(11):8939–49. doi: 10.3390/cimb45110561 (PMC10670264; doi:10.3390/cimb45110561)

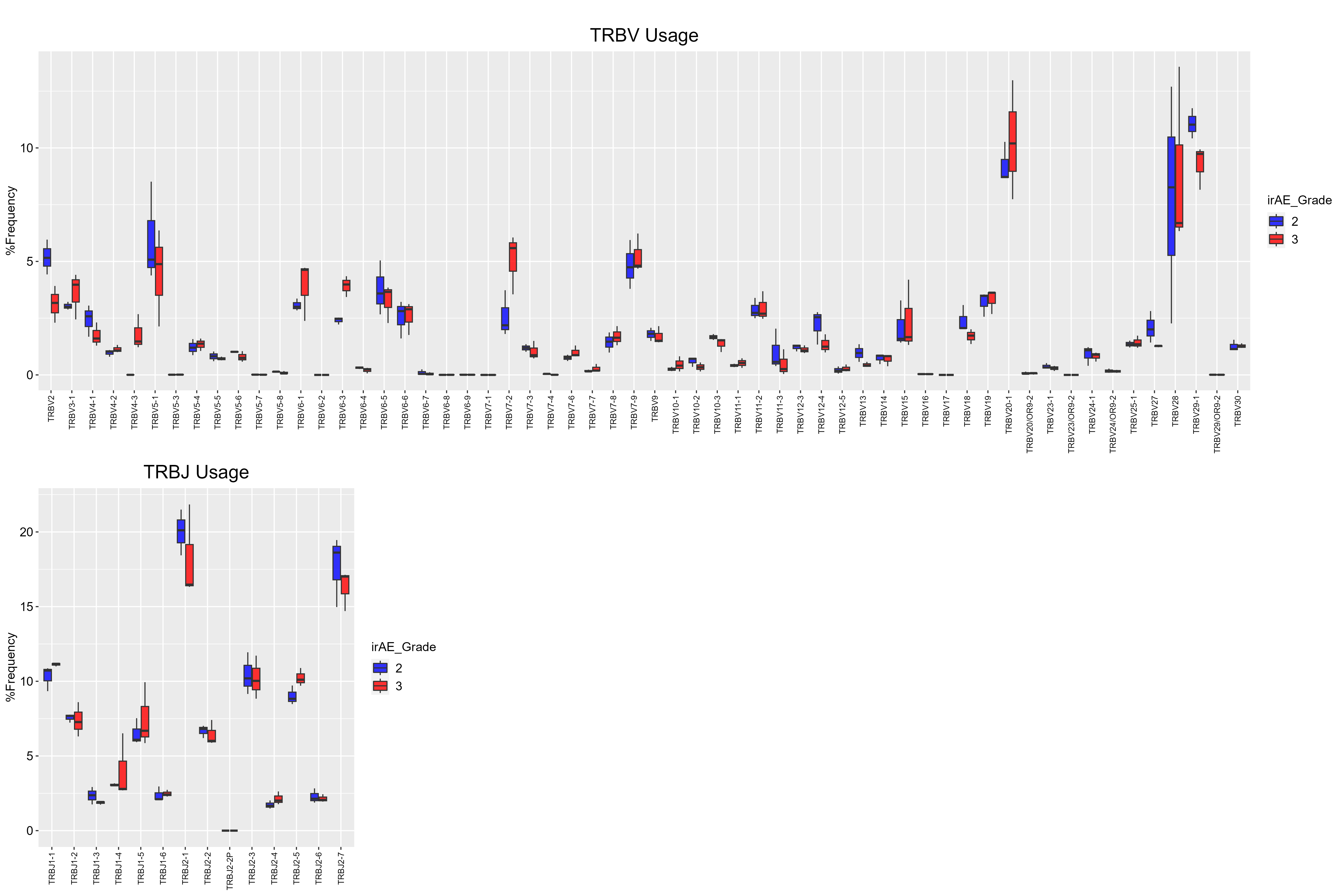

Supplement: Supplementary file 1 [file cimb-45-00561-s001.zip › FigureS1_TRB_Usage.tiff]
